# Dataset of Sentinel-1 SAR and Sentinel-2 RGB-NDVI imagery

**DOI:** 10.1016/j.dib.2024.111160

**Published:** 2024-11-20

**Authors:** Ahmed Alejandro Cardona-Mesa, Rubén Darío Vásquez-Salazar, Luis Gómez, Carlos M. Travieso-González, Andrés F. Garavito-González, Esteban Vásquez-Cano, Jean Pierre Díaz-Paz

**Affiliations:** a Faculty of Engineering, Politécnico Colombiano Jaime Isaza Cadavid, 48th *Av*, 7-151, Medellín, Colombia; b Electronic Engineering and Automatic Control Department, IUCES, Universidad de Las Palmas de Gran Canaria, Juan de Quesada 30, Las Palmas de Gran Canaria, Spain; c Signals and Communications Department, IDeTIC, Universidad de Las Palmas de Gran Canaria, Juan de Quesada 30, Las Palmas de Gran Canaria, Spain

**Keywords:** Sentinel, Synthetic aperture radar (SAR), Speckle, Deep learning, Supervised learning, Vegetation index

## Abstract

This article presents a comprehensive dataset combining Synthetic Aperture Radar (SAR) imagery from the Sentinel-1 mission with optical imagery, including RGB and Normalized Difference Vegetation Index (NDVI), from the Sentinel-2 mission. The dataset consists of 8800 images, organized into four folders—SAR_VV, SAR_VH, RGB, and NDVI—each containing 2200 images with dimensions of 512 × 512 pixels. These images were collected from various global locations using random geographic coordinates and strict criteria for cloud cover, snow presence, and water percentage, ensuring high-quality and diverse data. The primary motivation for creating this dataset is to address the limitations of optical sensors, which are often hindered by cloud cover and atmospheric conditions. By integrating SAR data, which is unaffected by these factors, the dataset offers a robust tool for a wide range of applications, including land cover classification, vegetation monitoring, and environmental change detection. The dataset is particularly valuable for training machine learning models that require multimodal inputs, such as translating SAR images to optical imagery or enhancing the quality of noisy data. Additionally, the structure of the dataset and the preprocessing steps applied make it readily usable for various research purposes. The SAR images are processed to Level-1 Ground Range Detected (GRD) format, including radiometric calibration and terrain correction, while the optical images are filtered to ensure minimal cloud interference.

Specifications Table

**Table:** 

Subject	*Earth-Surface Processes, Applied Machine Learning, Global and Planetary Change*
Specific subject area	*Applied remote sensing, Synthetic Aperture Radar (SAR) imagery, optical imagery*
Data format	*Filtered and processed images in GeoTIFF (.tif) format.*
Type of data	*Folders of Images*
Data collection	*The images were obtained from Sentinel-1 Synthetic Aperture Radar, in Level 1 Detected High-Res Dual-Pol (GRD-HD) with VV and VH polarizations, and from Sentinel-2 optical sensors. Random locations were used, and every image is 512 pixels in height and 512 in width.*
Data source location	*The data were collected from Google Earth Engine using random geographical coordinates from around the world*
Data accessibility	Repository name: Mendeley Data Data identification number: DOI 10.17632/xjcr5k4c9t.1 Direct URL to data: https://data.mendeley.com/datasets/xjcr5k4c9t/1 Instructions for accessing these data: Go to the URL, navigate to the root folder and its subfolder, and download them. Consider the dataset description.
Related research article	

## 1 Value of the Data

• The collection provides images of various random locations worldwide, containing the Synthetic Aperture Radar (SAR) imagery at GRD-HD level with both VV and VH polarizations from Sentinel-1, as well as optical RGB and Normalized Difference Vegetation Index (NDVI) data derived from Sentinel-2. These images (SAR VV, SAR VH, RGB and NDVI) are properly rescaled and co-registered.
• This dataset's value is multifaceted, offering many applications in remote sensing and machine learning. Firstly, researchers can use this dataset to develop and evaluate algorithms for image quality enhancement, such as advanced speckle noise reduction techniques in SAR imagery. Additionally, the dataset is ideal for creating image translation models that convert SAR data into optical (RGB) or NDVI images, which is crucial for agricultural studies, environmental monitoring, and climate change analysis. It can also be used to train deep neural networks for land cover classification, multi-temporal change detection, and natural disaster analysis. Lastly, the well-organized and co-registered structure of the images facilitates integration with other geospatial datasets, enabling users to conduct broader and more comprehensive research on various geographical and environmental phenomena.
• The dataset contains 8800 images divided into 4 folders (VV, VH, RGB and NDVI), with each folder containing 2200 images. Each image has dimensions of 512 × 512 pixels. The images were obtained with low cloud and water percentage to ensure heterogeneity.
• The methodology used in this dataset can be replicated with other regions, a larger amount of data or other search criteria, since the platform used to download the images allows the addition of different filters with the properties provided by the Sentinel data catalog.

## 2 Background

The development of this dataset was driven by the need to address the limitations inherent in optical satellite imagery, which is often affected by atmospheric conditions like cloud cover, obscuring critical surface features. This limitation poses significant challenges for continuous Earth observation, particularly in tropical and equatorial regions, where cloud cover is prevalent. SAR technology, with its ability to penetrate clouds and operate under all weather conditions, offers a robust alternative for obtaining consistent and reliable surface data.

The dataset presented here combines Sentinel-1 SAR imagery with Sentinel-2 optical data (RGB and NDVI) to create a comprehensive resource for research in remote sensing and machine learning. The theoretical basis for this integration lies in the complementary nature of SAR and optical imagery: while SAR provides structural and textural details, optical data adds spectral richness and visual interpretability. This multimodal dataset facilitates various applications, including land cover classification, vegetation monitoring, and environmental change detection. The dataset aims to enhance the accuracy and generalizability of models used in Earth observation studies.

## 3 Data Description

The dataset presented in this work is a comprehensive collection of satellite images that combine Synthetic Aperture Radar (SAR) data from Sentinel-1 with optical imagery from Sentinel-2, including RGB and NDVI data. The primary motivation for creating this dataset arises from the limitations inherent in passive optical sensors, particularly their susceptibility to cloud cover and atmospheric conditions, which can obscure the Earth's surface and hinder accurate analysis. SAR, on the other hand, uses microwave signals that can penetrate clouds and work in all weather conditions, making it an invaluable tool for continuous and reliable Earth observation. A labeled dataset with only Sentinel-1 SAR images to train despeckling models was proposed in [1].

The dataset contains a total of 8800 images, organized into four distinct folders: SAR_VV, SAR_VH, RGB, and NDVI. Each folder comprises 2200 images with dimensions of 512 × 512 pixels. The SAR_VV and SAR_VH folders contain images from Sentinel-1 dual-polarization radar, which captures data in vertical transmit and receive (VV) and vertical transmit and horizontal receive (VH) polarizations. These polarizations are particularly useful for analyzing surface roughness, soil moisture, and vegetation structure. The RGB folder contains true color images derived from Sentinel-2, which are crucial for visual interpretation and analysis of land cover. The NDVI folder includes NDVI images calculated from Sentinel-2 near-infrared and red bands, providing essential data for vegetation health assessment.

The images in this dataset are named using a consistent numerical naming convention, ensuring that images corresponding to the same geographic location across different modalities (VV, VH, RGB, NDVI) can be easily identified and cross-referenced. This consistency is vital for tasks such as data fusion, where information from different sensors is combined to create more comprehensive analytical models.

**Fig. 1:**
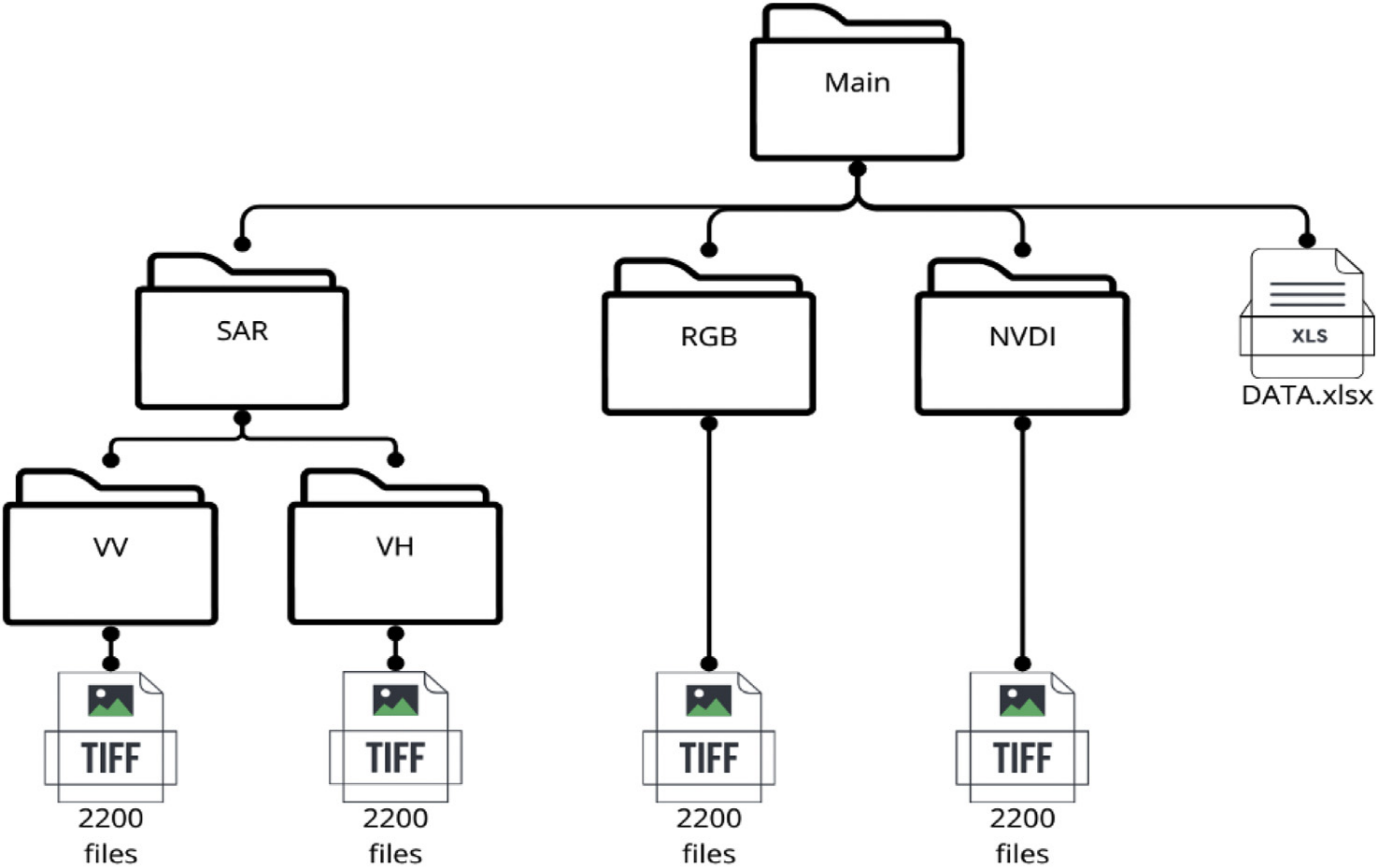
Structure of the dataset (main folder and subfolders).

The structure of the dataset is designed to facilitate ease of use and integration into various research workflows. The main directory is divided into four subfolders, each corresponding to one of the image types (SAR_VV, SAR_VH, RGB, NDVI). This organization allows users to easily navigate and access the specific data they need for their analyses. Fig. 1 illustrates the folder structure, highlighting the clear separation of data types.

Additionally, the dataset includes metadata that describes the acquisition parameters for each image, such as the date of capture, geographic coordinates, and sensor specifications. This metadata is crucial for understanding the context of the images and for conducting temporal analyses or comparing data across different regions or time periods.

**Fig. 2:**
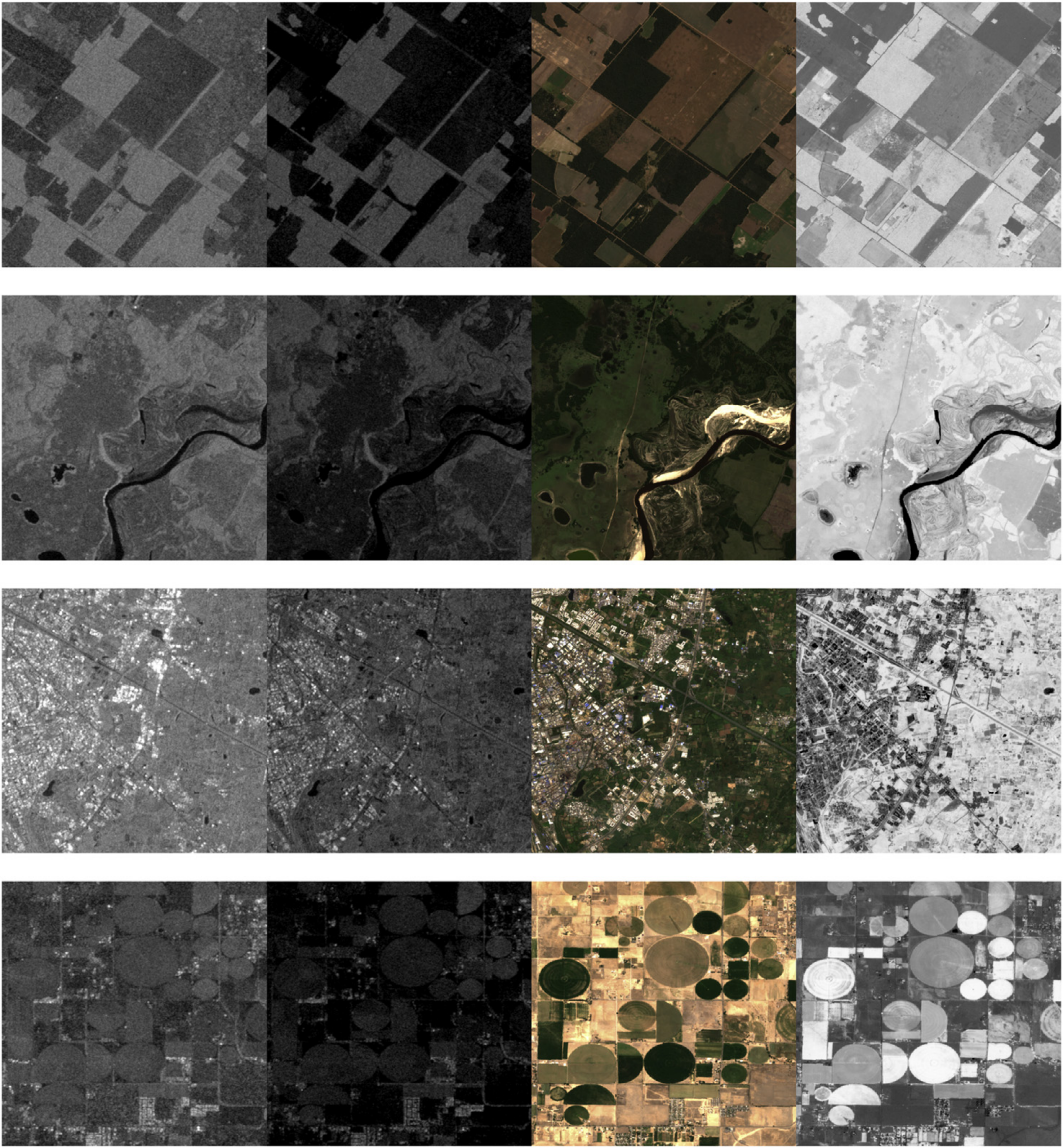
Images of the dataset. From top to bottom: four different locations. From left to right: SAR VV, SAR VH, RGB, and NDVI.

Fig. 2 provides a visual representation of the dataset, showcasing images from four different geographic locations. Each row in the figure corresponds to a distinct location, and the columns display the various types of imagery available for that location: SAR VV, SAR VH, RGB, and NDVI. This figure illustrates the diversity and complementary nature of the dataset, where SAR and optical images are aligned and presented side by side, enabling a comprehensive analysis of surface characteristics from different perspectives.

The images were selected and processed to ensure high quality and reliability. For the optical images, a cloud masking algorithm was applied to minimize the presence of clouds and atmospheric disturbances, ensuring that the underlying land surface is clearly visible. The SAR images were processed to Level-1 Ground Range Detected (GRD) format, which includes radiometric calibration and terrain correction (orthorectification). These preprocessing steps are essential for making the data ready for immediate use in various applications, from machine learning model training to geospatial analyses.

One of the key strengths of this dataset is its global coverage and the heterogeneity of the regions represented. The images were obtained using random geographic coordinates spanning a wide range of environments, from tropical rainforests to arid deserts, and from mountainous regions to coastal areas. This diversity makes the dataset particularly valuable for developing generalized models that can be applied to different geographical contexts. By including images from various regions and seasons, the dataset offers a rich resource for studying environmental processes on a global scale.

## 4 Experimental Design, Materials and Methods

### 4.1 Radar imagery

Synthetic Aperture Radar (SAR) is a special type of radar that generates images using microwave pulses which interact with objects on Earth's surface and then are backscattered to the remote sensor [2,3]. This technology is particularly advantageous in remote sensing because it can penetrate clouds and work effectively regardless of weather conditions or daylight availability. SAR systems, such as those on the Copernicus Sentinel-1 satellites, operate using a dual-polarization C-Band radar instrument at 5.5405 GHz, enabling the capture of detailed surface information in both VV and VH polarizations [4].

The Sentinel-1 mission, designed as a two-satellite constellation with Sentinel-1A launched in 2014 and Sentinel-1B in 2016, provides continuous and reliable imagery of Earthʼs surface. This capability is enhanced with the upcoming launch of Sentinel-1C in 2024 [5,6]. The SAR imagery collection from Sentinel-1 includes Ground Range Detected (GRD) scenes, which are processed using the Sentinel-1 Toolbox to achieve radiometric calibration, terrain correction (orthorectification), and other preprocessing steps [7,8]. These preprocessing steps are crucial for ensuring the accuracy and usability of the SAR data [9]. The SAR imageries collection provided by Sentinel-1 uses a dual-polarization C-Band radar instrument at 5.5405GHz. Some of the technical specifications of the Sentinel-1 images used in this dataset are listed in Table 1, including mission details, sensor characteristics, and processing levels. These specifications underscore the advanced capabilities of the Sentinel-1 mission in providing high-quality SAR imagery for diverse scientific and practical applications.

**Table 1:** Technical specifications of Sentinel-1 images.

Item	Description
Mission	Sentinel 1A-1B
International Partner	ESA
Altitude/Inclination	693 km/98.2°
Band	C (5.4GHz)
Beam Mode	Interferometric Wide Swath (IW)
Resolution	10 × 10 m
Revisit period	12 days (using together A and B, 6 days)
Processing Level	Level-1 Ground Range Detected (GRD)
Polarizations	VV - VH

SAR imagery is invaluable for a wide range of applications, including monitoring land use and land cover changes, detecting deforestation, assessing soil moisture, and supporting disaster management efforts such as flood and earthquake response. The high-resolution data provided by SAR can also be used for urban planning, infrastructure monitoring, and environmental conservation. The daily updates of the SAR collection in platforms like Google Earth Engine make it an accessible and up-to-date resource for researchers and practitioners in various fields [10].

### 4.2 Optical imagery

Sentinel-2 is a European wide-swath, high-resolution, multi-spectral imaging mission. This mission carries an optical instrument payload that samples 13 spectral bands, the orbital swath width is 290 km. The Sentinel-2 mission consists of two identical satellites, Sentinel-2A and Sentinel-2B, that were launched using the European VEGA launcher. The Multi Spectral Instrument (MSI) that collects the data works passively, by collecting sunlight reflected from the earth. New data is acquired by instrument as the satellite moves along its orbital path, the information collected is separated into two focal plane assemblies with the help of the MSI [11]. Some specifications of the sentinel-2 mission are shown in Table 2.

**Table 2:** Technical specifications of Sentinel-2 images.

Item	Description
Mission	Sentinel 2A-2B
International Partner	ESA
Altitude/Inclination	786 km/98.62°
Sensor	Multi Spectral Instrument (MSI)
Resolution	10 m - 20 m - 60 m
Revisit period	5 days at the Equator
Bands	The 13 spectral bands of Sentinel-2 range from the Visible (VNIR) and Near Infra-Red (NIR) to the Short Wave Infra-Red (SWIR)

The collection of images provided by the data catalog includes 13 bands. The 'B2′ (Blue), 'B3′ (Green), and 'B4′ (Red) bands are used to obtain the true color image. Additionally, the 'B8′ band is used as Near Infrared (NIR) to obtain the NDVI image using Eq. (1) (See [12,13])

(1) $$NDVI=\frac{\left(NIR-RED\right)}{\left(NIR+RED\right)}$$

This dataset's availability in Google Earth Engine allows for easy access and manipulation, enabling users to apply various filters and preprocessing steps to tailor the data to their specific research needs. The combination of Sentinel-2′s high-resolution optical imagery with the robust capabilities of Sentinel-1′s SAR data makes this dataset a powerful tool for a wide range of scientific and practical applications, from agricultural monitoring to climate change studies.

### 4.3 Search algorithm

**Fig. 3:**
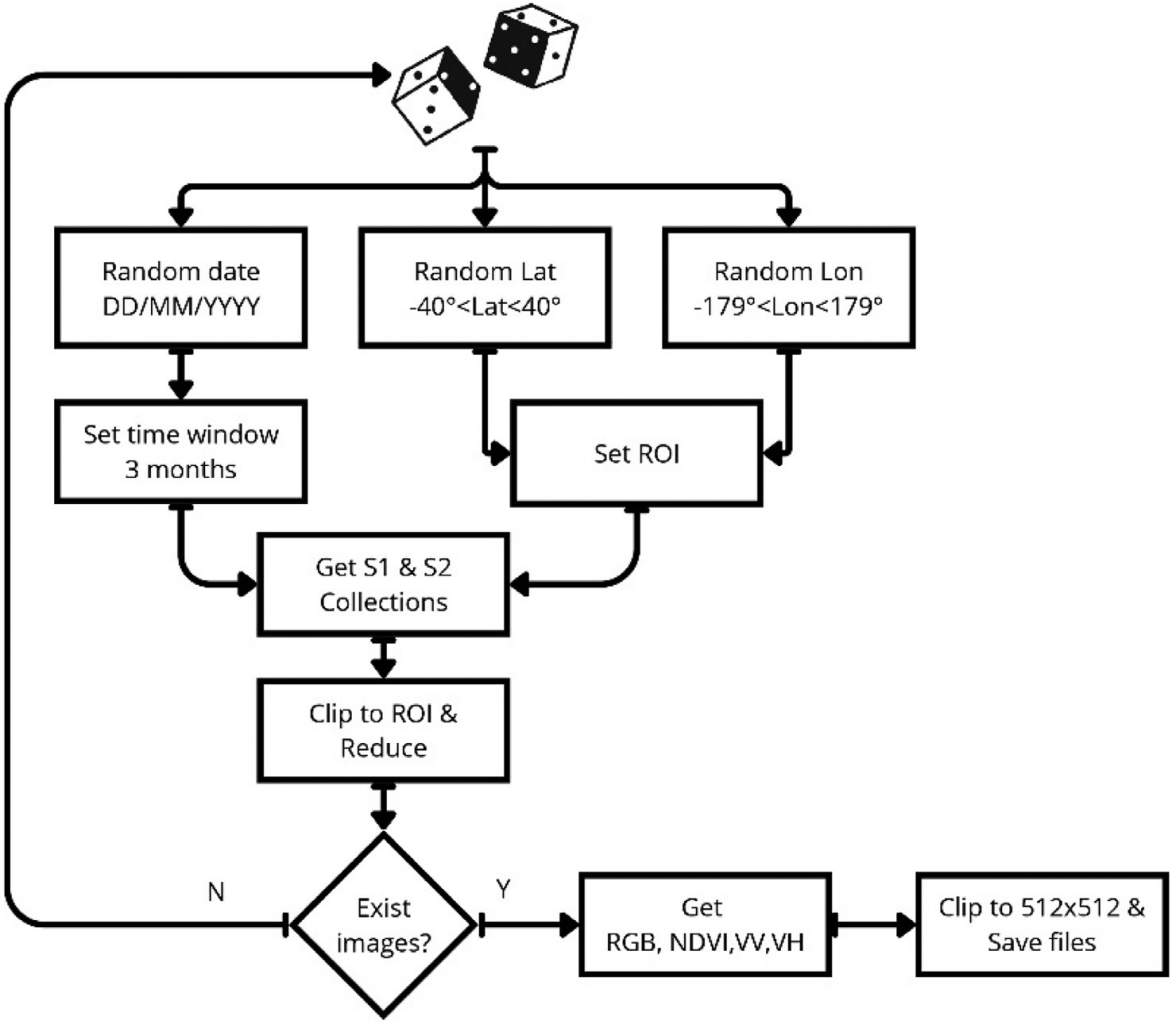
Search algorithm.

Aiming to obtain images from Google Earth Engine with heterogeneous regions and locations useful for studying tropical environments, a search algorithm in Python is used. First, a random scene of 0.1°x0.1°, with latitude between 40°S and 40°N WGS84 and dates between January 1, 2017, and March 1, 2024, is set. Then, the respective collections of Sentinel-1 and Sentinel-2 images that satisfy the optical and radar criteria from TABLE are obtained. A single image is generated for each collection using the median reducer and clip methods. The scene is accepted into the dataset if the previous search is successful for both Sentinel-1 and Sentinel-2 sources, meaning both images exist. Next, the NDVI is calculated as a normalized difference between the B8 and B4 bands from the Sentinel-2 image, the RGB image is obtained by stacking the B4, B3, and B2 bands from the Sentinel-2 image, and the VV and VH images are obtained by extracting the respective polarization. The images are clipped to 512 × 512 pixels and stored as GeoTIFF files to preserve the geographical information. This search was repeated until 2200 scenes were included in the dataset. The process is illustrated in Fig. 3.

Table 3 summarizes the specific optical and radar criteria applied during the data selection process for this dataset. These criteria were meticulously defined to ensure the acquisition of high-quality images that meet the stringent requirements necessary for accurate analysis and model development. For the optical imagery, factors such as cloud cover, snow presence, and water percentage were carefully controlled to minimize obstructions and enhance the clarity of surface features. On the radar side, parameters like polarization, revisit time, and resolution were selected to optimize the capture of detailed surface information across diverse environments. By adhering to these criteria, the dataset provides a robust and reliable foundation for a wide range of remote sensing applications.

**Table 3:** Optical and radar criteria.

Optical criteria	CLOUDY_PIXEL_PERCENTAGE	5
	SNOW_ICE_PERCENTAGE	5
	WATER_PERCENTAGE	40
	VEGETATION_PERCENTAGE	20
**Radar criterion**	orbitProperties_pass	DESCENDING

The code used in the previous sections is available on https://github.com/Andres01032002/Dataset-of-Sentinel-1-SAR-and-Sentinel-2-NDVI-Imagery, and the dataset with the structure described in Fig. 1 is available on https://data.mendeley.com/datasets/xjcr5k4c9t/1.

## Limitations

• The size of the images is 512 × 512 pixels, so the models trained by using this dataset must fit this size. If larger images are used, the model has to be applied by patches.
• The SAR images used are Level 1 GRD-HD. If raw images or lower levels are needed, the dataset is not suitable and another methodology must be used, since GEE does not provide lower level imagery.
• The geographic and temporal coverage of the dataset, while diverse, is also constrained by the random selection criteria. Although the dataset includes images from various regions and seasons, it does not systematically cover all possible environments or temporal conditions.
• The reliance on cloud-free optical imagery limits the datasetʼs usability in regions with persistently high cloud cover, such as equatorial rainforests or certain coastal areas.
• The dataset is primarily focused on Earth surface processes and may not be directly applicable to studies that require data from other domains, such as oceanography or atmospheric sciences.

## Ethics Statement

The authors confirm that, after reading the ethical requirements for publication in Data in Brief, the current work does not involve human subjects, animal experiments, or any data collected from social media platforms.

## Credit Author Statement

**Rubén Darío Vásquez-Salazar:** Conceptualization, Methodology, Writing - Original Draft; **Ahmed Alejandro Cardona-Mesa:** Methodology, Writing - Original Draft; **Luis Gómez:** Conceptualization, Methodology, Formal Analysis, Writing - Review & Editing; **Carlos M. Travieso-Gonzalez:** Conceptualization, Methodology, Formal Analysis, Writing - Review & Editing; **Andrés F. Garavito-González:** Software, Investigation, Resources, Writing - Original Draft; **Esteban Vásquez-Cano:** Software, Investigation, Resources, Writing - Original Draft. **Jean Pierre Díaz-Paz**: Conceptualization, Software, Investigation, Resources, Writing - Original Draft.

## Data Availability

Mendeley DataDataset of Sentinel-1 SAR and Sentinel-2 NDVI Imagery (Original data).

## References

[1] Vásquez-Salazar R.D., Cardona-Mesa A.A., Gómez L., Travieso-González C.M., Garavito-González A.F., Vásquez-Cano E. (2024). Labeled dataset for training despeckling filters for SAR imagery. Data Br..

[2] Cesare Mondini A., Guzzetti F., Chang K.-T., Monserrat O., Ranjan Martha T., Manconi A. (2021). Landslide failures detection and mapping using Synthetic Aperture Radar: Past, present and future. Earth-Sci. Rev..

[3] European Space Agency (ESA), “Synthetic Aperture Radar (SAR),” 2024. [Online]. Available: https://www.esa.int/SPECIALS/Eduspace_Global_ES/SEMVKXF64RH_0.html. [Accessed 12 august 2024].

[4] Bakon M., Teixeira A.C., Pádua L., Morais R., Papco J., Kubica L., Rovnak M., Perissin D., Sousa J.J. (2024). Synthetic aperture radar in vineyard monitoring: examples, demonstrations, and future perspectives. Remote Sens..

[5] Torres R., Davidson M., Geudtner D. (2020). IGARSS 2020 - 2020 IEEE International Geoscience and Remote Sensing Symposium.

[6] European Space Agency (ESA), “Sentinel-1,” 2024. [Online]. Available: https://www.esa.int/Applications/Observing_the_Earth/Copernicus/Sentinel-1. [Accessed 12 august 2024].

[7] Kuntla S.Kiran (2021). An era of Sentinels in flood management: Potential of Sentinel-1, -2, and -3 satellites for effective flood management. Open Geosci..

[8] Google Inc., “Sentinel-1 Algorithms,” 2024. [Online]. Available: https://developers.google.com/earth-engine/guides/sentinel1. [Accessed 12 august 2024].

[9] Navacchi C., Cao S., Bauer-Marschallinger B., Snoeij P., Small D., Wagner W. (2023). Utilising Sentinel-1’s orbital stability for efficient pre-processing of radiometric terrain corrected gamma nought backscatter. Sensors.

[10] Tsokas A., Rysz M., Pardalos P.M., Dipple K. (2022). SAR data applications in earth observation: an overview. Expert Syst. Appl..

[11] Kokhanovsky A., Gascoin S., Arnaud L., Picard G. (2021). Retrieval of snow albedo and total ozone column from single-view MSI/S-2 spectral reflectance measurements over Antarctica. Remote Sens..

[12] Yang K., Luo Y., Li M., Zhong S., Liu Q., Li X. (2022). Reconstruction of Sentinel-2 image time series using Google earth engine. Remote Sens..

[13] Lasaponara R., Abate N., Fattore C., Aromando A., Cardettini G., Di Fonzo M. (2022). On the use of Sentinel-2 NDVI time series and Google earth engine to detect land-use/land-cover changes in fire-affected areas. Remote Sens..

